# Dental Caries Status of Children and Adolescents in West Africa—A Literature Review

**DOI:** 10.3390/healthcare13090961

**Published:** 2025-04-22

**Authors:** Wai-Lam Chan, Hei-Yu Wong, Rowenna Yue, Duangporn Duangthip, Phoebe Lam

**Affiliations:** 1 Faculty of Dentistry, The University of Hong Kong, Hong Kong; jcwl@connect.hku.hk (W.-L.C.); haileyw@connect.hku.hk (H.-Y.W.); yuekawan@connect.hku.hk (R.Y.); 2 College of Dentistry, The Ohio State University, Columbus, OH 43210, USA; duangthip.2@osu.edu

**Keywords:** adolescents, caries experience, caries prevalence, child, children, dental caries, dmft, DMFT, literature review, oral health statistics, West Africa

## Abstract

**Objectives:** Dental caries is one of the most prevalent oral diseases worldwide, including in Africa. The aim of this article was to provide a comprehensive review of dental caries status of children and adolescents living in the West African region. **Methods:** Articles that fulfilled the study selection criteria were identified through systematic search in electronic databases (EMBASE and MEDLINE). Titles and abstracts were examined manually to screen for articles that study the caries prevalence and experience of children under the age of 18, and only English publications published from the years 2015 to 2024 were included. Publications that include participants with special healthcare needs were excluded. Relevant data related to caries prevalence and severity of participants below 18 were extracted with a standardized spreadsheet. **Results:** Out of 1288 studies, a total of 18 studies were included. Among the 16 countries in West Africa, only 3 countries (19%) including Ghana, Nigeria, and Senegal met the inclusion criteria, with a majority (15/18, 78.9%) focusing on Nigerian children and adolescents. The dmft scores of the included studies ranged from 0.06 to 3.04, and the DMFT scores ranged from 0.02 to 2.65. It is worth noting that dmft/DMFT scores across different countries were collected with a high heterogeneity in study design, and were thereby not directly comparable. **Conclusions:** The majority of the included studies were conducted in Nigeria. There are significant variations in caries prevalence and caries experience among children and adolescent in West Africa. Further research on oral health surveillance in West Africa is needed.

## 1. Introduction

Oral health is a critical component contributing to an individual’s overall health and well-being [1]. It holds particular importance for children as it allows them to eat, speak, and socialize without discomfort [2], thereby playing a significant role in their growth and development [3]. Significant correlations were found between the number of decayed teeth and oral symptoms, which in turn affects the individual’s oral health-related quality of life [4]. The oral health status comprises untreated caries of deciduous and permanent teeth, severe periodontal disease, edentulism (total tooth loss), and cancer of the lip and oral cavity. These contribute to a remarkable public health problem on an international level, namely the deteriorating health of individuals [5].

Dental caries, in particular, is a multifactorial disease resulting from the interaction of dietary sugars, oral bacteria, and the host’s dentition over time [6,7]. It is characterized by the demineralization of teeth, leading to cavities and, if left untreated, potentially more severe dental and systemic health issues [8]. More than 30% of the world’s population have untreated dental caries, and it remains one of the most important oral health diseases due to its negative impacts on growth, cognitive development, and quality of life [9]. As reported in the Global Status Report on Oral Health 2022, due to their large populations, the South-East Asia region and Western Pacific region were reported to have the highest case numbers among the World Health Organization (WHO) regions, and among the four other WHO regions, namely the African, Americas, European, and Eastern Mediterranean regions, the Africa region recorded the highest number of cases at 480 million in 2019 [5]. The WHO also reported a global prevalence of caries in deciduous teeth at 43%, with the African region recording a caries prevalence of 30% in deciduous teeth in 2019 [10].

In some African countries, limited access to oral health services may result in untreated oral diseases, significantly impacting people’s quality of life. Data from the WHO reflected that in 2019, an estimated 28.5% of individuals over the age of 5 in the WHO African region had untreated caries in their permanent teeth, while 38.6% of children aged 1–9 were affected by untreated caries [10].

West Africa is home to over 460 million people, including 29 million children under the age of five [11,12]. Nigeria, the most populous country in West Africa, has over 232 million residents [13]. The West African region, as defined by the United Nations, includes the following countries: Benin, Burkina Faso, Cabo Verde, Côte d’Ivoire, The Gambia, Ghana, Guinea, Guinea-Bissau, Liberia, Mali, Mauritania, Niger, Nigeria, Senegal, Sierra Leone, and Togo [14]. Historically, many of these countries were colonized by France and other Western countries in the early 1900s.

Data from indicators including population figures, Human Development Index (HDI), income level, Gross Domestic Product (GDP), percentage of government expenditure on education in GDP, water fluoridation status, dentist to population ratio, and the prevalence of untreated caries of deciduous teeth in children 1–9 years (%) were extracted from the websites of the WHO, United Nations Data, United Nations Human Development Reports, and the World Bank Group. A summary of socioeconomic data and oral health-specific statistics for each country is presented in Table 1. Most West African countries belong to low and low–middle-income countries, which reflects the USD 1145 or lower gross national income (GNI) per capita in 2023 for low-income countries, and USD 1146–4515 GNI per capita in 2023 for low–middle-income countries. GNI is the sum of the incomes of residents in an economy over a period of time [15]. However, West Africa is considered one of the least committed regions in Africa when it comes to reducing inequality [16], which can have implications for government spending on healthcare services. In Oxfam’s Commitment to Reducing Inequality Index (CRI), which measures governments commitments to free public services including education, healthcare and social protection, fair tax, labor rights, and reasonable wages, West African countries such as Nigeria (163rd), Liberia (157th), and Sierra Leone (156th) were ranked among the lowest among 164 countries [16].

A systematic review and meta-analysis on caries prevalence in 12-year-olds in Africa revealed that from 2000 to 2020, only decayed, missing, and filled teeth (DMFT) data from 3 countries including Nigeria (0.60), Burkina Faso (0.70), and Gambia (2.27) were available out of the 16 countries in the West African region [17]. An updated dental caries status in both primary and permanent dentition is required for oral health policymakers in planning caries preventive measures for children in West Africa.

This study aimed to summarize the prevalence and severity of dental caries among children and adolescents under 18 years of age in West African countries over the past decade by reviewing currently available studies. The null hypothesis was that there would be no difference between the caries prevalence and severity between countries in West Africa and when compared with other parts of the world.

**Table 1:** Socioeconomic and demographic indicators, water fluoridation status, and dentists per 10,000 population figures for each West African country.

Country	Population in 2023 (million) [18]	HDI in 2022 [19]	Income [18] *	GDP in 2023 (million) [20]	% Government Expenditure on Education [21]	Water Fluoridation (mg/L) [22] ^	Dentists per 10,000 Population [23]
Benin	14.1	0.504	LM	19,673.3	3.4% in 2022	0.54	0.05 in 2023
Burkina Faso	23	0.438	L	20,324.6	5.3% in 2022	0.05–0.24	0.06 in 2023
Cabo Verde	0.5	0.661	LM	2587.3	4.7% in 2022	Not available	0.15 in 2023
Côte d’Ivoire	31.2	0.534	LM	78,788.8	3.5% in 2022	Not available	0.14 in 2023
The Gambia	2.7	0.495	L	2339.9	2.7% in 2023	Not available	0.03 in 2022
Ghana	34.1	0.602	LM	76,370.4	2.9% in 2022	0–20.7	0.07 in 2022
Guinea	14.4	0.471	LM	23,612.3	2.0% in 2022	Not available	0.03 in 2022
Guinea-Bissau	2.2	0.483	L	1966.5	Not available	Not available	0.10 in 2023
Liberia	5.5	0.487	L	4240	2.4% in 2023	Not available	0.02 in 2022
Mali	23.8	0.41	L	20,661.8	4.0% in 2022	0.2–1.7	0.01 in 2022
Mauritania	5	0.54	LM	10,651.7	2.3% in 2022	Not available	0.31 in 2022
Niger	26.2	0.394	L	16,819.2	4.1% in 2022	4.8–6.6	0.01 in 2022
Nigeria	223.8	0.548	LM	363,846.3	0.3% in 2022	0.01–7.16	0.23 in 2022
Senegal	17.8	0.517	LM	30,848.3	5.6% in 2022	1.1–7.4	0.11 in 2023
Sierra Leone	8.5	0.458	L	3809.8	6.8% in 2023	0.02–0.85	0.02 in 2022
Togo	9.3	0.547	L	9171.3	3.8% in 2022	0.15–1.39	0.02 in 2022

HDI, Human Development Index; GDP, Gross Domestic Product; *, income levels: L, low (USD 1145 or lower GNI per capita in 2023); LM [13], lower middle (USD 1146–4515 GNI per capita in 2023); ^, the fluoride status in these countries has been investigated based on the concentration of fluoride in drinking groundwater sourced from rocks or minerals [22].

## 2. Materials and Methods

Studies were included based on the systematic search of the electronic databases EMBASE and MEDLINE. Oral Health [MeSH Terms] AND Children [MeSH Terms] AND West Africa [MeSH Terms] were used as keywords. Titles and abstract were examined manually, and only English publications were included.

Publications were included if they satisfied the following requirements:

- Cross-sectional studies;
- Primary data analyses;
- Papers published from 2015–2024;
- Examination of children, under 18 years of age, in West African countries;
- Caries prevalence was expressed as the percentage of children under 18 years of age affected by dental caries, expressed as the mean decayed, missing, and filled teeth (dmft and/or DMFT scores) score, depending on children’s dental development.

The screening of the retrieved articles through title and abstracts was completed independently by 3 reviewers (J.W.C., R.K.Y., H.H.W.). Publications that included populations that were inconsistent with the current study selection were excluded. This involved groups such as children with an HIV infection and children with special education needs, as they were not considered representative of the general population. Studies utilizing randomized controlled clinical trials were also excluded. Full papers were retrieved to extract data if possible, and replication of publications were excluded. Conflicts were resolved through discussion or consulting the 4th reviewer (P.P.L.).

Data were extracted in accordance with the following: (i) countries; (ii) sources of information (year of publication); (iii) area/detail of study; (iv) year of survey; (v) sampling method; (vi) caries diagnostic criteria; (vii) sample size; (viii) age of participants; (ix) caries prevalence (expressed in per cent); and (x) mean dmft/DMFT score.

## 3. Results

A total of 1288 publications were found in the literature search on EMBASE and MEDLINE conducted on 14 June 2024. Out of these, 1259 articles were excluded based on their titles and abstracts as they were deemed not to meet the inclusion criteria. The excluded articles included studies that solely reported caries prevalence in adults over 18 years of age, studies focused on caries status in children with an HIV infection, reports where caries status was not presented in terms of dmft/DMFT, and data collected from countries outside of Western Africa.

**Figure 1:**
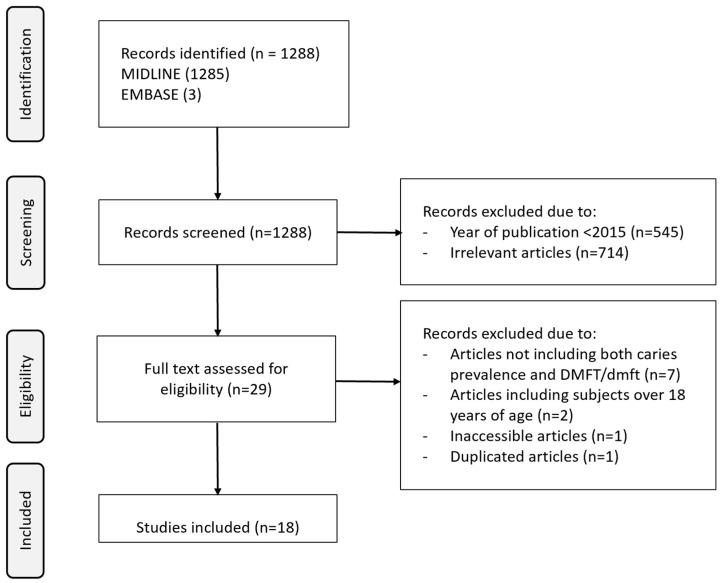
Flow diagram for identification, screening, and study selection.

A total of 29 full papers were retrieved and reviewed, of which 11 studies were excluded because they failed to include both caries prevalence and dmft/DMFT data. Ultimately, only 18 studies conducted in Ghana, Nigeria, and Senegal were included [24,25,26,27,28,29,30,31,32,33,34,35,36,37,38,39,40,41]. The majority of the included studies (78.9%) were from Nigeria. A total of 39% (7/18) of the included studies were conducted in urban area, 56% (10/18) from semi-urban or suburban areas, and 17% (3/18) from rural areas. Half (50%, 9/18) of the included studies collected data through schools, and more than one-third (34%, 6/18) of the studies were collected through local government areas. The majority (84%, 15/18) of the included studies used the WHO as a diagnostic method, and multistage sampling was the most common sampling method employed. Sample size ranged from 50 to 2107. A flow chart illustrating the identification and study selection process is presented in Figure 1, while socioeconomic data and oral health-specific statistics for each country are summarized in Table 1. The specifics of the caries experience as reported in the studies are outlined in Table 2.

Two recent studies in Ghana were identified. The reported caries prevalence varied widely from 13.3% to 40.4% [24,25]. One study that was carried out in urban schools reflected a mean DMFT of 0.27 with a caries prevalence of 13.3% [24]. Another study that was carried out in Urban Accra and Rural Kpando reflected a caries prevalence of 38.9% to 40.4% among children aged 3–4 and 6–13 years old. Their mean dmft/DMFT scores ranged from 0.49 to 1.48 [25].

Sixteen studies reported the caries prevalence and severity in Nigeria [26,27,28,29,30,31,32,33,34,35,36,37,38,39,40,42]. Infants and children aged from 0.5 months to 15 years old exhibited a wide range of caries prevalence, varying from 3.4% to 96%. The dmft and DMFT scores also showed significant variability, ranging from 0.06 to 3.04 and 0.02 to 2.65, respectively. Among the studies conducted in Nigeria, 5 studies in urban areas reported higher mean dmft or DMFT scores [27,28,31,37,38] compared to the other 11 studies conducted in semi-urban, suburban, or rural areas. For example, in a study focusing on pre-school children in five local government areas in Lagos State, Nigeria, the mean dmft score was reported as 3.04 [38], which was the highest among studies conducted in various parts of Nigeria and other West African countries.

Only one study reported the caries status in Pikine, Senegal, revealing a caries prevalence of 64.8% and a caries experience (expressed as the mean mixed DMF score) of 2.5 [41].

## 4. Discussion

This review article presented an overview of oral health conditions, focusing on caries experiences and prevalence in children under the age of 18, as indicated by mean dmft/DMFT scores. It summarized data from 16 West African countries over the past decade. The available data showed that caries prevalence in West Africa, based on dmft scores from the included studies, ranged from 0.06 to 3.04, while DMFT scores ranged from 0.02 to 2.65. Additionally, caries prevalence in Ghana, Nigeria, and Senegal ranged from 13.3% to 40.4%, 3.4% to 96%, and 64.8%, respectively. Surprisingly, among the 29 full papers retrieved and reviewed, only 18 studies conducted in three countries, namely Ghana, Nigeria, and Senegal were included. This highlights the pressing need for more research to be conducted in 13 other West African countries in order to provide up-to-date oral health information representing the general population in West Africa.

In comparison to caries prevalence in East Africa over the past 20 years, among children and adolescents under the age of 18, caries prevalence was reported at 45.7% [43]. In South Africa, the percentage of untreated caries in children aged 1–9 years old was 41.0% [44]. Likewise, the Central African Republic (40.0%) [45] in the Central African region and Cameroon (39.2%) [46] in the North African region exhibited caries prevalence rates comparable to those in East and South Africa, which are higher than the majority of the caries prevalence rates reported in this current study. The wide range of caries prevalence across West African countries in this study suggests the presence of potential inter- and intra-country variations in oral health outcomes.

The potential variation in caries prevalence across countries may be associated with the influence of socioeconomic factors, including population size, GDP, HDI, and the proportion of government spending on education. Most West African countries are classified as low or low–middle-income countries, with a significant proportion of them having a prevalence of untreated caries in deciduous teeth of children aged 1–9 years exceeding 40% in 2019 [47]. Nigeria, with an HDI of 0.548, well below the world average of 0.739, presented a caries prevalence up to 96% [38]. A study by Nazir et al. in 2021 found a statistically significant negative correlation between the percentage of GDP allocated to education and caries experience, suggesting that government expenditure on education can impact oral health behaviors, such as oral hygiene practices, and subsequently influence caries prevalence and risks [48]. This underscores the importance of investing in education as a means to improve oral health outcomes in the population, and implies that social factors such as health, education, and standard of living, as reflected in the HDI, as well as other socioeconomic factors such as GDP, could potentially influence the prevalence of caries in children.

A study from Ghana showed discrepancies in caries prevalence and dmft/DMFT between urban and rural areas [25], suggesting that oral health status could be influenced by geographical locations and urbanization. Similarly, in countries or regions with a high Gini index, such as South Africa, a significant variability in caries experiences among children and adolescents were also identified [49]. However, due to a lack of sufficient data, it is challenging to definitively conclude the existence of such a correlation.

This literature review underscores the importance of focusing on dental caries prevention in children and adolescents in West Africa based on the findings in [50]. Fluoride is recognized as a crucial element in preventing dental caries [50]. The WHO recommends that fluoride concentrations in drinking water should not exceed 1.5 ppm (parts per million) to balance the risks of dental fluorosis and the benefits of preventing dental caries [51]. However, the availability of national fluoridation programs in West African countries is not well documented. The fluoride status in these countries has been investigated based on the concentration of fluoride in drinking groundwater sourced from rocks or minerals [22]. The British Fluoridation Society reported that Libya is the only African country with a government water fluoridation program, with 22% of the population consuming optimally fluoridated water based on data predating 2003 [52]. Water fluoridation has been scientifically proven to be effective in preventing dental caries [53]. Nevertheless, data on fluoride concentrations in drinking water across Africa remains limited, and further research is needed to explore its relationship with caries prevalence in the region. Additionally, access to clean water is a crucial factor in implementing water fluoridation programs. According to the Progress On Drinking Water, Sanitation, and Hygiene in Africa 2000–2020 report, a significant portion of the population in urban and rural areas in Africa lack access to safely managed drinking water [54]. Therefore, it is essential to consider both the fluoridation of drinking water and the availability of clean water in efforts to prevent dental caries effectively.

In addition, it is notable that most West African countries have a low dentist-to-population ratio ranging from 0.01 to 0.23 dentists per 10,000 people between 2022 and 2023 [23]. These ratios are considerably lower than the global average of 3.3 dentists per 10,000 people in 2022, as reported by the WHO. The availability of dentistry personnel, not only in terms of overall ratios but also the distribution between rural and urban areas, can have a significant impact on the oral health of individuals. For instance, in Ghana, despite having the highest dentist-to-population ratio among West African countries, there was a notable disparity in DMFT scores between children living in Urban Accra and Rural Kpando [42]. Further research should be carried out to assess the distribution of dentistry personnel in various areas, as the accessibility of dental care providers may play a crucial role in addressing oral health issues in West Africa. Understanding and addressing the disparities in the availability and distribution of dental professionals can help improve oral health outcomes across the region.

Diversity in the study design, diagnostic criteria, sample groups, and potential influence of socioeconomic factors have led to a wide range of caries prevalence rates in the studies included in this review. To facilitate a more effective comparison and analysis of caries prevalence in the African region, it is recommended that more research focusing on oral health surveillance in Western Africa be conducted. This would help provide more standardized and comparable data for a better understanding of the oral health landscape in the region.

However, one limitation of this review is the exclusion of non-English articles. As English is not an official language in some Western African countries such as Togo, Benin, and Senegal, research conducted in these countries outside of the English medium might not be available. Studies that were published in other languages, such as Arabic and French, were excluded. Moreover, the inclusion criteria of this review were confined to articles published from 2015 to 2024. More up-to-date information could be available but was excluded in our review. In addition, the West African region, as defined by the United Nations, includes Benin, Burkina Faso, Cabo Verde, Côte d’Ivoire, The Gambia, Ghana, Guinea, Guinea-Bissau, Liberia, Mali, Mauritania, Niger, Nigeria, Senegal, Sierra Leone, and Togo [14]. In the African Development Bank Group, Mauritania is included in the North African region [55]. This shows a discrepancy in the definition of the West African region between different organizations. Lastly, literature reviews are not easily repeatable, and search strategies are less vigorous than those of systematic reviews. Publication bias could also arise leading to an overestimation of the effects from positive findings.

Through the comparison of existing studies, we hope to encourage more observational studies on oral health condition in these countries, thereby allowing more thorough investigations on the effects of the country’s socioeconomic status, demographics, and general oral hygiene habits. With more data and evidence becoming available, more attention could be drawn to improving the dental health of the West African population, improving oral health awareness, and strengthening action plans to alleviate oral health burdens. The implementation of preventive measures, interventions including water fluoridation, general oral health education, dental system establishment, and the provision of dental outreach services are encouraged. Lastly, through the results of this literature review, we hope to provide insights on evidence-based dentistry that can aid clinicians to be well informed on the latest oral health status of children and adolescents in West Africa and ensure the effectiveness of subsequent interventions, and facilitate cooperation between national and international policymakers to improve global oral health.

## 5. Conclusions

In conclusion, 3 out of 16 West African countries, namely Ghana, Nigeria, and Senegal, reported their respective oral health status data for children and adolescents under 18 for the past 10 years. The majority of the included studies (78.9%) were from Nigeria. The caries prevalence in West Africa in terms of dmft scores of the included studies ranged from 0.06 to 3.04, and DMFT scores ranged from 0.02 to 2.65. More research focusing on oral health surveillance in Western Africa is needed.

## Figures and Tables

**Table 2:** Caries prevalence and caries experience of children and adolescents (<18 years) in Western Africa.

Country	First Author Year	Area	Place	Sampling Method	Diagnostic Criteria	Sample Size	Age (years)	% Caries ^1^ dmft/DMFT > 0 ^2^ dt/DT > 0	dmft (SD)	DMFT (SD)
Ghana	Blankson 2022 [24]	Urban	School	NA	WHO	1118	9–16	13.3 ^2^	-	0.27 (0.76)
	Peters 2022 [25]	Urban and	School	Convenience	WHO	156	3–13	40.4 ^1^	0.97 (1.65)	0.83 (1.39)
		Rural				157		38.9 ^1^	1.48 (2.56)	0.49 (0.98)
Nigeria	Folayan 2015 [26]	Suburban	LGAs	Multistage	WHO	497	0.5–5	6.60 ^1^	0.15	-
	Olatosi 2015 [27]	Urban	Clinic	Simple Random	WHO	302	0.5–5	21.2 ^1^	0.735 (2.07)	-
	Chukwumah 2016 [28]	Urban	School	Multistage	WHO	1790	12–15	21.9 ^1^	-	12 y/o: 1.76 (1.72) 13 y/o: 1.77 (1.12) 14 y/o: 2.37 (1.81) 15 y/o: 2.65 (1.94)
	Kolawole 2016 [29]	Semi-urban	LGAs	Multistage	WHO	992	1–12	10.5 ^1^	0.22 (0.8)	0.04 (0.3)
	Folayan 2017 [30]	Semi-urban	LGAs	Cluster	WHO	601	5–12	14.8 ^1^	0.27 (0.85)	0.06 (0.38)
	Tobin 2017 [31]	Urban and Rural	School	Stratified cluster	WHO	50	5–6	14 ^1^	0.3	-
						50	12	4 ^1^	-	0.04
	Adeniyi 2017 [32]	Semi-urban	School	Multistage	NA	414	8–12	21 ^2^	-	0.442 (1.078) *
	Oyedele 2018 [33]	Sub-urban	School	Multistage	WHO	2107	8–16	12.2 ^2^	0.06	0.16
	Akinyamoju 2018 [34]	Rural	School	Multistage	WHO	778	11 ± 1.8	12.2 ^1^	-	0.2 (0.7) *
	Kolawole 2019 [35]	Suburban	LGAs	Multistage	NA	495	6–12	14.9 ^2^	0.27 (0.82)	0.07 (0.39)
	Folayan 2019 [36]	Suburban	LGAs	Multistage cluster	WHO	370	0.5–5.9	4.86 ^2^	0.14 (0.8)	-
	Okolo 2022 [37]	Urban	School	Multistage	WHO	366	10–12	25.4 ^2^	-	0.6 (1.3) *
	Olatosi 2022 [38]	Urban	School	Multistage	WHO	273	1–5	96 ^1^	3.04 (2.28)	-
	Folayan 2022 [39]	Semi-urban	LGAs	Multistage	WHO	1326	6–11	5.2 ^1^	0.08 (0.457)	0.02 (0.159)
	Adeniyi 2023 [40]	Semi-urban	NA	Multistage	NA	1411	6–12	3.8	0.09 (0.475)	0.02 (0.196)
Senegal	Dieng 2020 [41]	Suburban	NA	Multistage cluster	WHO	315	3–9	64.8	-	2.5 (2.7) *

dmft: decayed, missing, and filled teeth index for the primary dentition; DMFT: decayed, missing, and filled teeth index for the permanent dentition; dt, decayed primary teeth; DT, decayed permanent teeth; ^1^ dmft/DMFT > 0: decayed, missing, and filled teeth index for the primary dentition or permanent dentition larger than zero; ^2^ dt/DT > 0: decayed teeth index for the primary dentition or permanent dentition larger than zero; LGAs, local government areas; NA, not available; *, unspecified dmft or DMFT (SD).

## Data Availability

As the data used in this study were generated from published sources, all information is publicly available.
